# Significant pain reduction and improved functional outcome after surgery for displaced midshaft clavicular fractures

**DOI:** 10.1186/s13018-015-0336-z

**Published:** 2015-12-24

**Authors:** Lars Eden, Dirk Ziegler, Fabian Gilbert, Kai Fehske, Annabel Fenwick, Rainer H. Meffert

**Affiliations:** Department of Trauma, Hand, Plastic and Reconstructive Surgery, Julius Maximilian University, Oberduerrbacher Straße 6, 97080 Wuerzburg, Germany

**Keywords:** Clavicular fracture, TEN, AS clavicle plate LCP, Reconstruction plates

## Abstract

**Purpose:**

Displaced midshaft clavicular fractures can be treated conservatively as well as operatively by titan elastic nail (TEN) or plate fixation. This survey was performed to evaluate the clinical results of each treatment method and elaborate advantages or possible complications of each modality.

**Methods:**

Between 2008 and 2013, 102 patients were prospectively included in our study—37 patients for conservative treatment with a rucksack bandage for 4 to 6 weeks, 41 patients for plate osteosynthesis, and 24 for intramedullary stabilization with TEN. Disabilities of the Arm, Shoulder and Hand (DASH), Constant Murley Score (CMS), and visual analog scale (VAS) for pain and function as well as time of invalidity were recorded over a 1-year period.

**Results:**

The clinical data collected reveals that all three different therapies lead to good or excellent clinical results after 1 year. However, one can observe advantages of operative treatment in comparison to conservative therapy in some characteristics.

**Conclusion:**

Our data shows that there are several indications where operative treatment has advantages compared to conservative treatment. In special fracture types (Robinson 2B1), TEN gives the best results. Plate fixation is extraordinarily sufficient in pain reduction within the first 5 weeks and indicated in more-part fractures (Robinson 2B2). Nevertheless, conservative treatment is always a good and promising way to treat clavicular fractures, so that individual indications and thorough patient informative talks are inevitable.

## Introduction

Fractures of the midshaft clavicle account for approximately 4–8 % of all fractures and are thereby a frequently seen injury especially in young and active men [1, 2]. Three different standard treatment options are offered: conservative treatment with a sling or a rucksack bandage, intramedullary stabilization with a titan elastic nail (TEN), and open reduction and fixation with a plate (Fig. 6).

Neer’s data from the 1960s with a non-union rate less than 1 % has traditionally been the basis for the recommendation of conservative treatment [1]. Surveys in the 1990s revealed a significantly higher non-union rate for displaced fractures of 15 % and more than 30 % unsatisfied patients treated conservatively [3]. These results were underlined by the work of McKee in 2006 [4]. In 2007, a prospective randomized Canadian multicenter study showed that plate fixation is superior to conservative treatment mainly using non-locking LCDC plates [5]. By 2008, 26 % of all midshaft clavicular fractures were already treated operatively in Germany [6] mainly using reconstruction plates. We, however, observed a high rate of implant failure and less biomechanical stability using reconstruction plates [7]. Since then, the new plates (Locking compression plate (LCP) anterior-superior clavicle plate, Fa. DePuy Synthes) have been used in our clinic. Valid data concerning the clinical application of those plates is not available up to now.

Furthermore, the indications and clinical results of TEN are not well examined. In a meta-analysis, Zlowodzki elaborated the TEN to have good clinical results with a significantly lower rate of non-unions than conservative treatment [8], but prospective randomized surveys are not available.

To our experience, patients with a clavicle fracture are exceptionally active in terms of sport and eager to go back to work and sport as quickly as possible. To our knowledge, the current literature does not offer precise answers to how long disability is to be expected after injury.

Thus, we started a study in the year 2008 comparing the three mentioned groups over a 1-year period. Especially in the first 6 weeks, we collected data on a weekly basis and recorded the time of absence from work.

## Methods

Between 2008 and 2013, 102 patients were prospectively included in our study. All patients had a displaced midshaft fracture of the clavicle accounting for Robinson type B 1 and 2 fractures [2]. Undisplaced (less than a shaft of displacement), open, or pathologic fractures, as well as an age below 15 or associated brain injury (grades II and III), were excluded from the survey. The ethical committee of the University of Wuerzburg gave an approval for the investigation (reference number 69/08).

After explaining the situation and mentioning all therapy options including complications, the preferred treatment was chosen in accordance with the patient. There was the choice of conservative treatment with a rucksack bandage for 4 to 6 weeks (37) or an operative procedure. Plate fixation was mainly performed in Robinson 2B2 fractures, whereas TEN was the preferred method in Robinson 2B1 fractures with an oblique or transverse fracture. Plate osteosynthesis was applied in 41 patients and intramedullary stabilization with TEN in 24 patients. We exclusively used the anterior-superior clavicle plate by DePuy Synthes with 6, 7, and 8 holes. Reconstruction plates were not used. After operation, we immobilized the shoulder in a Gilchrist bandage for 1–2 weeks; abduction and elevation >90° were restricted for 6 weeks as was contact sport for 3 months. TEN was applied in the technique described by Rehm and Jubel [9]. In 54 %, a closed reduction could be performed. The postoperative care was identical to that mentioned above for plate procedure. Implant removal was recommended for TEN 6 months after surgery.

For the clinical evaluation, we used a visual analog scale for pain (0–100) and function (0–100) and the Disabilities of the Arm, Shoulder and Hand Score [10, 11] and the Constant Murley Score using a flexibar (IsoForceControl® Ca. Medical Device Solutions AG, Oberburg, Switzerland) for strength measurement [12]. In the first 6 weeks, the visual analog scale (VAS) scores for pain and function were documented weekly mainly by telephone. We arranged appointments in our outpatient clinic for X-ray, Constant Murley Score (CMS), Disabilities of the Arm, Shoulder and Hand (DASH), and VAS at 6, 12, 26, and 52 weeks after injury.

Furthermore, complications and the time of absence from work were recorded.

### Statistical analysis

Statistical analysis was carried out using a two-tailed Mann-Whitney *U*-test (SPSS 21.0, IBM). Figures have been made by MS Excel for Mac 2011, version 14.4.6.

## Results

Group-specific characteristics like mean age and fracture classification are listed in Table 1. The groups are not completely equal. The average age differs slightly (41, 38, and 34 years). The reason might be that older patients more likely avoid an operation and therefor increase the age average to 41 years. The fracture type also differed significantly between the groups. Since Robinson 2B1 fractures were predominantly treated with TEN, multipart fractures and comminuted fractures (2B2) were mostly treated by plate fixation.

**Table 1:** Group characteristics including complications

	Non-op	Plate	TEN
Number	37	41	24
Age in years (stand. dev.)	41 (18)	38 (15)	34 (14.5)
Robinson classification 2B1/B2	24/13	14/27	17/7
Non-union (%)	2 (5.4)	0	1 (4.2)
Open reduction (%)		41 (100)	11 (46)
Other complications		1 lateral plate tear out after 4 weeks because of non-compliance	1 delayed union (healed with ultrasound), 2 lateral dislocations of the TEN, 1 prominent nail medially: required shortening in local anesthesia
Revision rate in % (major)		2.4	8.4 (4.2)

**Fig. 1:**
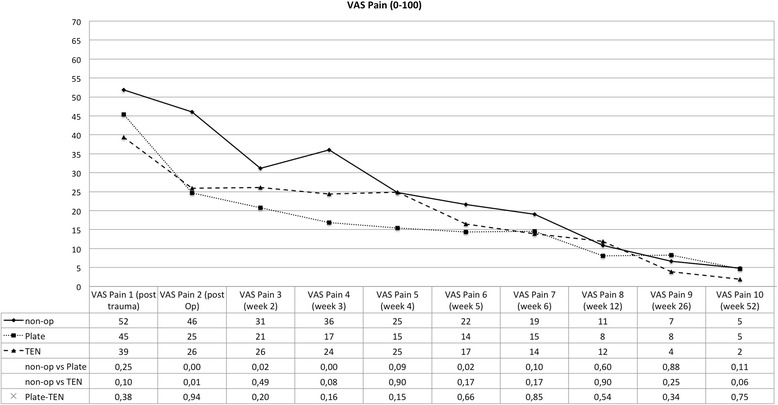
Visual analog scale for pain (0 = no pain; 100 = maximum pain); *p* values are listed below, and statistical significance was considered at *p* < 0.05

**Fig. 2:**
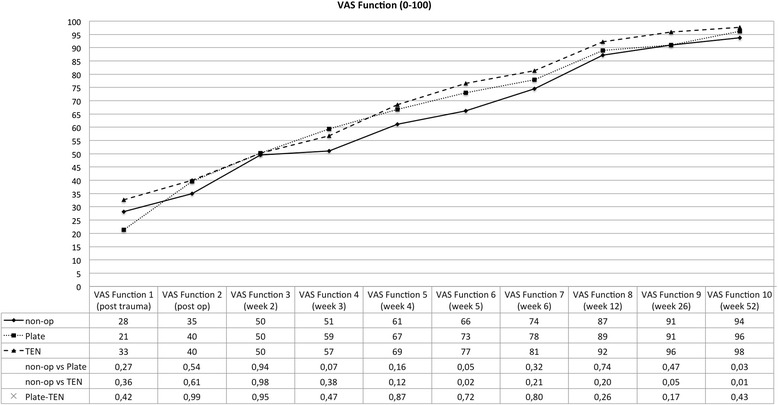
Visual analog scale for function (0 = no function; 100 = full recovery); *p* values are listed below, and statistical significance was considered at *p* < 0.05

**Fig. 3:**
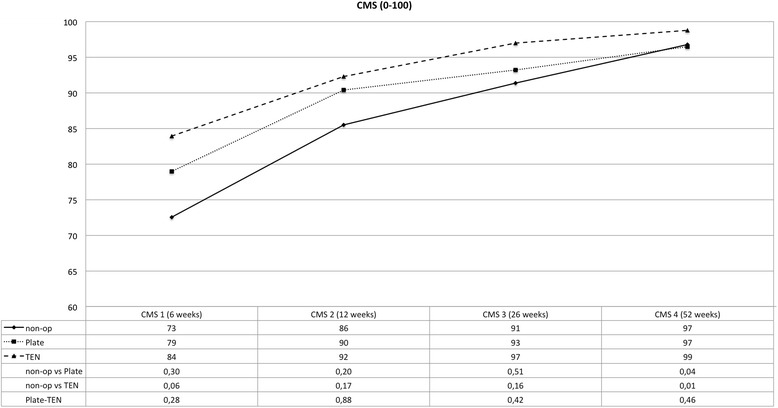
Constant Murley Score; *p* values are listed below, and statistical significance was considered at *p* < 0.05

**Fig. 4:**
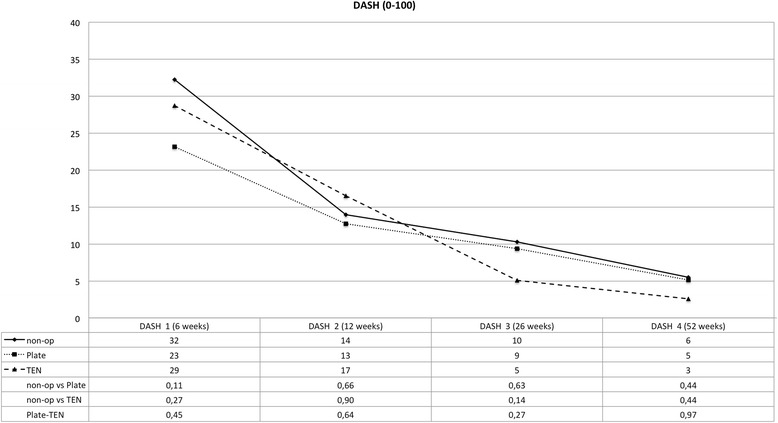
Disabilities of the Arm, Shoulder and Hand Score

The clinical data collected reveals that all three different therapies for displaced midshaft clavicular fractures lead to good or excellent clinical results after 1 year (see Figs. 1, 2, 3, and 4). However, one can observe advantages of operative treatment in comparison to conservative therapy for certain characteristics. Especially for pain reduction in the early postoperative phase, plate fixation is superior to conservative treatment. The VAS scores for pain are significantly lower for the plate group (*p* < 0.05), 1, 2, 3, and 5 weeks after operation. The TEN group shows significantly lower figures in terms of pain compared to conservative treatment 1 week after operation (see Fig. 1). The subjective function score (VAS) reveals significantly better results on average for TEN after 6 months and 1 year. The figures for plate fixation are lower but still significantly superior after 1 year compared to conservative treatment (see Fig. 2). The Constant Murley Score offers better results for TEN at all time points, being significant at 1 year after injury. Plate fixation is also significantly superior compared to conservative treatment after 1 year (see Fig. 3). The DASH scores do not show any differences between the groups examined (see Fig. 4).

**Fig. 5:**
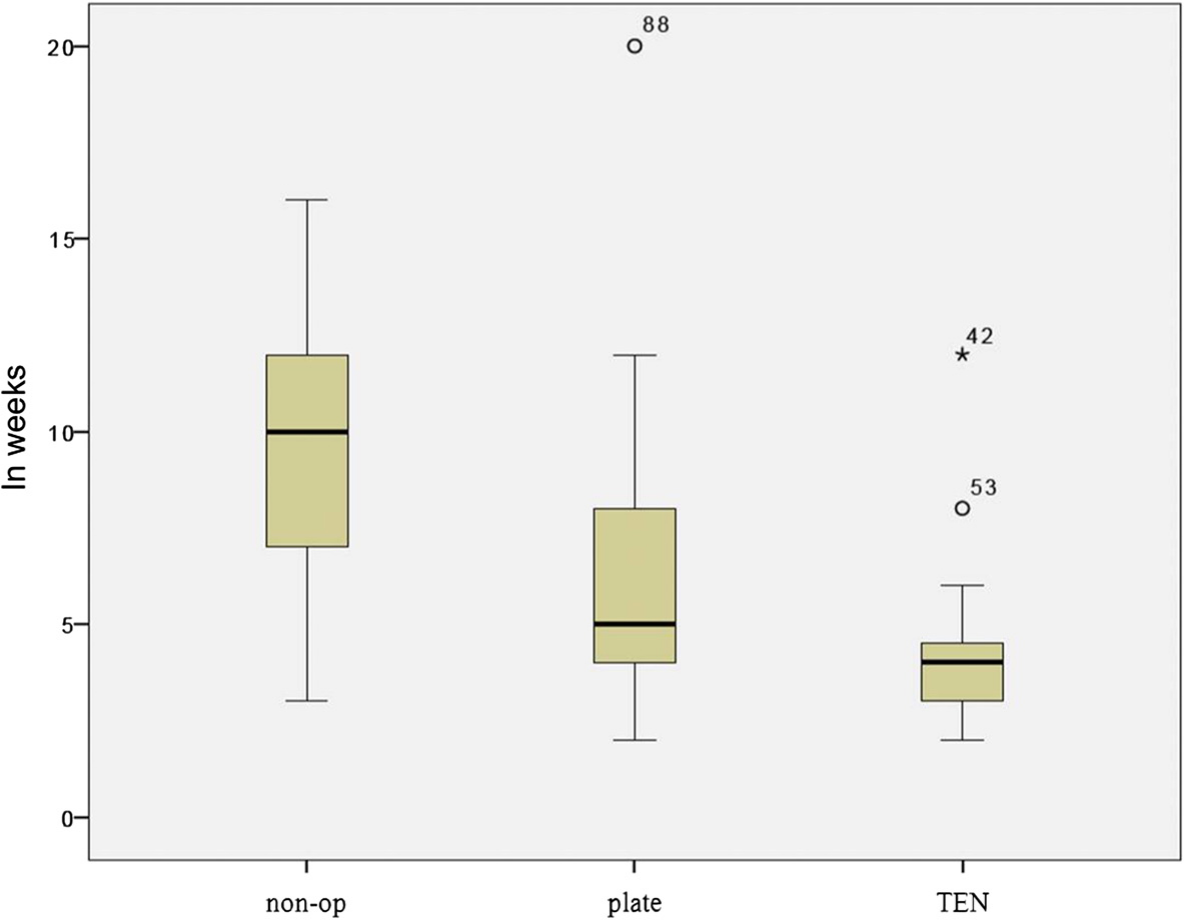
Time of absence from work in weeks with standard deviation (*box plot*) and median

Time of disability to work differs significantly between the groups. Operation significantly reduced the time from an average of 9.4 (non-op) to 5.6 weeks. Patients treated with TEN just had an average of 4.5 weeks until they returned to work, plate fixation 6.2 weeks. Both are significantly lower than conservative treatment (see Fig. 5).

**Fig. 6:**
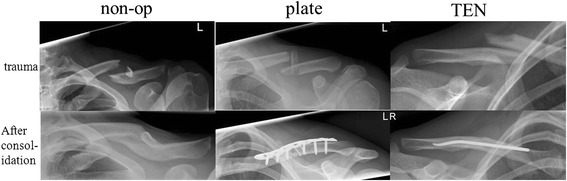
Example of the examined treatment methods: similar fractures treated conservatively, by plate and TEN

**Fig. 7:**
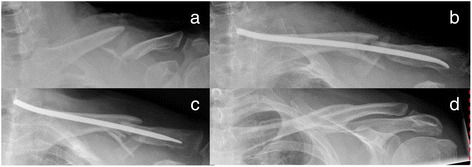
Robinson 2B2 fracture (**a**) treated with TEN (**b**); telescoping effect after 6 weeks at the medial side (**c**), after consolidation and implant removal (**d**)

Non-union is the most severe complication recorded. For plate fixation, all 41 patients treated healed within 6 months. One patient returned 4 weeks after the operation with a lateral tear out of the plate admitting that he had not followed the restrictions mentioned above. Two revisions with plate fixation were needed to achieve healing. No plate breakage or other implant failure was noticed. Some patients complained of local numbness around the incision made, which had mostly diminished at the time of the 1-year follow-up. Except in the one case mentioned above, no revision had to be performed. There were no other complications as hematoma or infection in the patients of the study. In the TEN group, one non-union was recorded requiring a revision with plate fixation and iliac crest interposition (Fig. 6). One patient showed delayed union, which was treated with ultrasound finally healing after 9 months. Two patients needed early implant removal because the nails dislocated dorso-laterally. The first patients treated with TEN mainly complained of prominent nails medially after tissue detumescence and telescoping (see Fig. 7). One patient needed shortening of the nail in local anesthesia. From there on, the nail was shortened to bone level thereby reducing this problem significantly.

Two non-unions were recorded using conservative treatment. One needed an operation with plate fixation and iliac crest interposition later. The other patient lacked complaints, so conservative treatment was continued.

## Discussion

Even though clavicular fractures are common in traumatology, precise recommendations for the treatment are still difficult and controversial. The “typical” patient is young, sportive and requests specific information about the different therapy options including risks, complications, outcome, and time to return to work and sport. Despite other clinical trials, there are still open questions to address the individual needs of the patient.

Neer’s work from the 1960s shows a very low rate of non-unions [1], being the basis for conservative treatment recommendations for a long period of time. Recently published works, however, reveal a significantly higher non-union rate and more unsatisfied patients in conservative compared to operative treatment [2–4]. A Canadian multicenter randomized trial showed plate fixation to be superior to conservative treatment in relation to non-union rate, patient satisfaction, and clinical scores (DASH, CMS) [5]. Smekal compared TEN to conservative treatment in a prospective non-randomized trial and recommends TEN for displaced simple oblique and transverse fractures [13]. Assessing the literature, there are strong statistical indications that an operation reduces the risk of non-union significantly [14, 15] and thereby improves the clinical outcome. However, an operation is more likely to cause complications in the beginning than conservative treatment and might need a second operation for implant removal.

Although our trial was not designed to randomize, we kept the preoperative consent open and allowed the patient’s decision to influence the treatment. Some felt strongly to stay without; others had recommendation from elsewhere and wanted surgery. We felt that most follow-up criteria are on a subjective basis and the patient’s treatment wish—no matter if surgery or not—has a positive effect on their evaluation.

Our data confirms that an operation and in our case especially plate fixation is the most secure way to achieve bone healing. The new stabilization systems (LCP, DePuy Synthes) are obviously stable enough to withstand the torsional and bending forces applied to the clavicle in vivo, corresponding to the results found in vitro [7]. The anterior position at the medial end and superior position at the lateral end might be of benefit for the biomechanical properties and requirements—without in vivo or in vitro proof so far. Reconstruction plates are no longer used in our department.

Another advantage of plate fixation that can be deduced from the study is the significant pain reduction immediately after operation. Up to 5 weeks after the operation, plate fixation consistently offers the lowest figures in terms of pain level. Patients treated with a plate returned 6.24 weeks in average after trauma to work which is significantly shorter in time than conservative treatment (9.36 weeks). On the other hand, 1 year after the operation, the VAS for pain and DASH show nearly the same results (see Figs. 1 and 4) for plate fixation and conservative treatment—questioning the necessity of an operation—even more so when a plate removal is performed because of hardware irritation or patient wish. This data is consistent with McKee’s meta-analysis published in 2012 [15]. CMS and VAS concerning function, however, favor plate fixation after 1 year (see Figs. 2 and 3).

Although TEN appears to have the best clinical outcome, some restrictions have to be pointed out. Closed reduction was only achieved in about half of our patients (54 %). The indications for TEN are limited and had to be clarified. Initially, we performed TEN also in Robinson 2B2 fractures, leading to telescoping effect and disturbing nails on the medial side and shortened clavicles (see Fig. 7). Lately, we restricted the indication to Robinson 2B1 fractures, thereby reaching excellent results. Throughout all statistics, TEN performed best—not always reaching statistical significance but still being conspicuous. A disadvantage is the obligatory removal after 6 months, which we do not recommend performing in local anesthesia. Absence from work averages 4.47 weeks, which is even lower than plate fixation and significantly less than conservative treatment (see Fig. 5). Jubel et al. 2003 already emphasized that TEN can be a good way to bring athletes back into their sport as quickly as possible [16], so that it seems reasonable that an early return to work is also possible. In summary, we agree with Smekal that TEN is a good and probably the best procedure treating short oblique and transverse dislocated midshaft clavicular fractures without extra fragments [13].

Conservative treatment always remains a good option in less displaced fractures and less active patients. More complicated fractures might be treated with surgical methods. The 1-year follow-up figures are very good. Non-union is more likely to occur in conservative treatment—not as often in our study as described in previous studies reaching up to 23 % [15]. But even when non-union occurs, it does not necessarily mean reduced function and pain. One of our patients was absolutely symptomless while having a radiographically proven non-union. On the other hand, one has to realize that a non-union operation with interposition of the iliac crest is much more demanding and complicated than initial treatment. Additionally, conservative treatment inevitably leads to malunion in the degree of initial displacement. Our data does not show that there are obvious limitations in function at a certain degree of shortening or displacement. For a precise analysis, though, CT analysis and comparison to the contralateral side are necessary, which was not performed in this study.

The study has some other limitations. We started to attempt a randomized trial but realized that it is not practicable to obtain the amount of patients needed. Most patients could not be convinced that fortune decides what kind of treatment will be performed. Due to recommendations of others or a fixed opinion of the patient, the open preoperative consent was much easier leaving the “last” of decision to the patient. Thus, the level of evidence is reduced and the conclusions drawn have lower value.

Due to our bad experience in the treatment of multifragment fractures, we do not recommend the use of TEN for these. Telescoping of the fracture may occur, and the nuchal end of the TEN causes soft tissue irritation (see Fig. 7).

Hardware removal is another important issue that has to be considered and discussed. All TEN patients received a removal after 6 months in general anesthesia. Patients with plate fixation were not reoperated for hardware removal until the 1-year follow-up. Probably some of the problems including pain and function deficit in this group are due to the plate. Patients described problems with safety belts or wearing rucksacks. In the group of conservative treatment, just one required operative revision because of a non-union. In older patients with an increased risk for anesthesia, this is definitely an important point to keep in mind.

A plus of the study is the consistent use of the same implants. All plate patients received an anterior-superior clavicle plate (Fa. DePuy Synthes) just differing in the amount of holes (6, 7, 8), while all TEN patients received a TEN (Fa. DePuy Synthes 2.0–3.5 mm). Clinical data with this new stabilization system is still rare. Our study shows that they are a good and reliable way to treat clavicular fractures with no implant failure.

In conclusion, our data shows that there are some indications in which operative treatment has advantages compared to conservative treatment. In special fracture types (Robinson 2B1), TEN produces excellent results especially in terms of reduction of disability and subjective (VAS) and objective shoulder scores (CMS) after 1 year for a selected fracture type. Plate fixation is extraordinarily sufficient in pain reduction within the first 5 weeks and indicated in more-part fractures (Robinson 2B2). Nevertheless, conservative treatment is always a good and promising way to treat clavicular fractures, so that individual indications and thorough patient informative talks are inevitable.

For further clarification, more randomized trials have to been performed in the future.
